# Clinical, biochemical and genetic profiles of patients with mucopolysaccharidosis type IVA (Morquio A syndrome) in Malaysia: the first national natural history cohort study

**DOI:** 10.1186/s13023-019-1105-6

**Published:** 2019-06-14

**Authors:** Huey Yin Leong, Nor Azimah Abdul Azize, Hui Bein Chew, Wee Teik Keng, Meow Keong Thong, Mohd Khairul Nizam Mohd Khalid, Liang Choo Hung, Norzila Mohamed Zainudin, Azura Ramlee, Muzhirah Aisha Md Haniffa, Yusnita Yakob, Lock Hock Ngu

**Affiliations:** 1 Genetics Department, Hospital Kuala Lumpur, Ministry of Health Malaysia, Jalan Pahang, 50586 Kuala Lumpur, Malaysia; 2 Unit of Molecular Diagnostics & Protein, Institute for Medical Research, Ministry of Health Malaysia, Kuala Lumpur, Malaysia; 3 Department of Paediatrics, Faculty of Medicine, University Malaya, Kuala Lumpur, Malaysia; 4 Paediatric Department, Hospital Kuala Lumpur, Ministry of Health Malaysia, Kuala Lumpur, Malaysia; 5 Ophthalmology Department, Hospital Selayang, Ministry of Health Malaysia, Selayang, Malaysia

**Keywords:** Natural history, Diagnosis, Mucopolysaccharidosis IVA, *GALNS*, Malaysia

## Abstract

**Background:**

Mucopolysaccharidosis IVA (MPS IVA) is an autosomal recessive lysosomal storage disease due to N-acetylgalactosamine-6-sulfatase (GALNS) deficiency. It results in accumulation of the glycosaminoglycans, keratan sulfate and chondroitin-6-sulfate, leading to skeletal and other systemic impairments. Data on MPS IVA in Asian populations are scarce.

**Methods:**

This is a multicentre descriptive case series of 21 patients comprising all MPS IVA patients in Malaysia. Mutational analysis was performed by PCR and Sanger sequencing of the *GALNS* gene in 17 patients.

**Results:**

The patients (15 females and 6 males) had a mean age (± SD) of 15.5 (± 8.1) years. Mean age at symptom onset was 2.6 (± 2.1) years and at confirmed diagnosis was 6.9 (± 4.5) years. The study cohort included patients from all the main ethnic groups in Malaysia – 57% Malay, 29% Chinese and 14% Indian. Common presenting symptoms included pectus carinatum (57%) and genu valgum (43%). Eight patients (38%) had undergone surgery, most commonly knee surgeries (29%) and cervical spine decompression (24%). Patients had limited endurance with lower mean walking distances with increasing age. *GALNS* gene analysis identified 18 distinct mutations comprising 13 missense, three nonsense, one small deletion and one splice site mutation. Of these, eight were novel mutations (Tyr133Ser, Glu158Valfs*12, Gly168*, Gly168Val, Trp184*, Leu271Pro, Glu320Lys, Leu508Pro). Mutations in exons 1, 5 and 9 accounted for 51% of the mutant alleles identified.

**Conclusions:**

All the MPS IVA patients in this study had clinical impairments. A better understanding of the natural history and the clinical and genetic spectrum of MPS IVA in this population may assist early diagnosis, improve management and permit timely genetic counselling and prenatal diagnosis.

**Electronic supplementary material:**

The online version of this article (10.1186/s13023-019-1105-6) contains supplementary material, which is available to authorized users.

## Introduction

Mucopolysaccharidosis type IVA (MPS IVA, OMIM #253000, Morquio A syndrome) is an autosomal recessive lysosomal storage disease. MPS IVA is characterized by a deficiency of the lysosomal enzyme N-acetylgalactosamine-6-sulfatase (GALNS), which is required for the degradation of the glycosaminoglycans (GAGs), keratan sulfate (KS) and chondroitin-6-sulfate (CS) [1]. This enzyme deficiency leads to an abnormal accumulation of KS and CS, and their excretion in the urine [1]. GALNS deficiency distinguishes MPS IVA, the more common form, from mucopolysaccharidosis type IVB in which beta-galactosidase activity is deficient [1].

Progressive accumulation of KS and CS primarily in cartilage and the extracellular matrix results in systemic skeletal dysplasia, which varies in severity but is present in all patients [2]. Clinical features of those with the classical phenotype include short stature, prominent forehead, short neck, pectus carinatum, kyphoscoliosis, genu valgum, hypermobile joints and cervical instability with spinal cord compression [2]. These abnormalities all restrict patient mobility and endurance [2].

Instability of the cervical spine due to odontoid process hypoplasia and ligamentous laxity may lead to dislocation and compression of the cervical cord, resulting in cervical myelopathy and paralysis [3]. The severe skeletal dysplasia also causes chest wall restriction, which can be exacerbated by respiratory muscle weakness associated with cervical myelopathy. Furthermore, laryngeal narrowing and tracheal and bronchial abnormalities cause airway obstruction [3, 4]. These result in dyspnoea, recurrent respiratory infections and sleep-disordered breathing and may progress to respiratory failure [5].

Additional symptoms of MPS IVA may include hearing loss, corneal clouding and heart valvular disease, among others [5]. Life expectancy varies by phenotype. Patients with severe disease may survive only to late childhood or adolescence, whereas patients with more attenuated forms may live to 20–40 years of age; occasionally, patients survive for more than 60 years [6, 7]. Death is usually due to respiratory failure or spinal cord compression [6].

MPS IVA is a rare disorder and, although reliable and consistent reports of global incidence are not available, national and regional estimates of birth prevalence range from 1 in 76,000 live births in Northern Ireland to 1 in 641,000 live births in Western Australia [8, 9]. Data for MPS IVA in Asian populations are scarce, but available estimates of birth prevalence include 1 in 500,000 live births in Japan and 1 in 304,000 live births in Taiwan [10, 11].

International guidelines recommend multiple approaches for the management of MPS IVA. These include surgery to address musculoskeletal manifestations, vaccination and/or prompt aggressive treatment for respiratory infections, cardiac valve replacement, and vision and hearing aids [12]. Enzyme replacement therapy (ERT) with recombinant elosulfase alfa is an emerging treatment for MPS IVA. In a phase 3 trial, weekly dosing with elosulfase alfa was found to improve endurance, as measured by the 6-min walk test (6-MWT), and reduce urine KS levels [13]. Treatment guidelines recommend initiating ERT as soon as the diagnosis of MPS IVA is confirmed [12].

While information on the natural history of MPS IVA in broad populations is available from large international studies, [14, 15] studies in Asian populations are scarce, although some data are available for Taiwan and Korea [16, 17]. The aim of this study is to document the natural history of MPS IVA and better understand the spectrum of disease in Malaysian patients.

## Patients and methods

The Malaysia Morquio A Programme (MyMAP) is a first multicentre attempt to describe the clinical, biochemical and genetic profiles of all MPS IVA patients in Malaysia. After securing informed consent from patients and/or their parents, all Malaysian patients with MPS IVA were enrolled from the genetics clinics of Hospital Kuala Lumpur, Hospital Pulau Pinang and University Malaya Medical Centre, Kuala Lumpur. Diagnosis of MPS IVA was confirmed by a clinical geneticist based on documented reduced GALNS activity in leukocytes and/or molecular analysis of the *GALNS* gene. This study was approved by the Medical Research and Ethics Committee, Ministry of Health, Malaysia.

Between November 2014 and November 2016, 21 patients from 16 families were enrolled, representing all the confirmed MPS IVA patients in Malaysia. None of the patients had received haematopoietic stem cell transplant or ERT at the time of recruitment. To calculate birth prevalence, estimates of total live births in Malaysia from the birth year of the youngest patient to that of the oldest (years 1985 to 2013) were obtained from the Department of Statistics Malaysia. Data were collected according to prespecified study procedures and it was anticipated that not all subjects would have completed all assessments. Patients’ medical records were retrospectively reviewed for medical history, clinical manifestations, radiology findings, surgical procedures and laboratory studies. Clinical data collected during the study period included height, weight and physical examination, such as general appearance, neurological examination and muscle strength. Muscle strength was graded according to the Medical Research Council’s muscle power scale [18]. For the purpose of this study, the patients were classified as severe phenotype if height measurements plotted below the 90th percentile in published growth charts for MPS IVA [19]. Endurance was assessed during the study period with a 6-MWT performed according to published guidelines [20].

The patients were referred to various clinical specialists during the study period and any results from these assessments were recorded from their medical records. Ophthalmological data collected included best corrected visual acuity (measured using the appropriate chart according to age), presence of corneal clouding, glaucoma, pigmentary retinopathy and optic disc changes from slit lamp examination, indirect ophthalmoscopy and cycloplegia refraction. Age-appropriate audiometry data on subjects’ hearing ability, standard 2-dimensional Doppler echocardiography data and spirometry parameters such as forced vital capacity (FVC) and forced expiratory volume in 1 s (FEV1) were recorded. The patients may not have attended all the assessments.

Results of leukocyte GALNS enzyme activity analysis, quantitative urine GAGs analysis, and qualitative urine KS and CS analysis, per local laboratory guidelines, were retrospectively obtained from medical records.

### Mutation analysis

Approximately 5 to 10 mL of peripheral blood was collected from both patients and their parents for molecular analysis of the *GALNS* gene at the Institute for Medical Research, Kuala Lumpur. Genomic DNA was extracted using the QIAcube system (Qiagen) and both the quantity and quality of extracted DNA were measured using a NanoDrop ND-1000 Spectrophotometer (NanoDrop). Primers were designed in-house to amplify all 14 coding exons and flanking intronic sequences of the *GALNS* gene (NM_000512.4). Amplification was performed using touchdown PCR protocol as described by Azize et al. [21]. Purification of PCR products and Sanger sequencing was performed as described by Abdul Wahab et al. [22].

Sequencing results were aligned to the *GALNS* gene reference sequence (NM_000512.4) using SeqScape software v.3.0 (Applied Biosystems) to identify DNA variants. All variants identified were compared against The Human Gene Mutation Database (HGMD) (http://www.hgmd.cf.ac.uk/ac/index.php) [23], ClinVar (https://www.ncbi.nlm.nih.gov/clinvar/) [24], Genome Aggregation Database (gnomAD) (http://gnomad.broadinstitute.org/) [25] and the *GALNS* Mutation Database (http://galns.mutdb.org/) [26]. Novel variants were further checked using variant data from both the 100 genomes of Singaporean Malays retrieved from the Singapore Sequencing Malay Project (SSMP) (http://phg.nus.edu.sg/StatGen/public_html/SSMP/SSMP_index.html) [27] and the 38 genomes of Singaporean Indians retrieved from the Singapore Sequencing Indian Project (SSIP) (http://phg.nus.edu.sg/StatGen/public_html/SSIP/supp_methods.html#) [28].

The pathogenicity of novel DNA variants was evaluated by using four in silico programs: MutationTaster2 (http://www.mutationtaster.org/) [29], FATHMM-XF (http://fathmm.biocompute.org.uk/fathmm-xf/) [30], Mendelian Clinically Applicable Pathogenicity Score (M-CAP) (http://bejerano.stanford.edu/mcap/) [31] and Condel (https://bbglab.irbbarcelona.org/fannsdb/) [32]. All novel mutations identified in this study were submitted to the *GALNS* Mutation Database. Parental samples were also tested for the presence of the mutations detected in their child.

Homology modelling was performed to examine the potential effects of the novel missense mutations on protein structure. The crystal structure of the human GALNS enzyme was obtained from the Protein Data Bank (https://www.rcsb.org/; PDB ID:4FDI) [33] and, using this structure as a template, we modelled the protein structures for novel missense mutations using SWISS-MODEL (https://swissmodel.expasy.org/) [34]. Visual comparison between wild type and mutant protein structures was carried out using PyMOL Molecular Graphics System version 2.1.1 (Schrödinger, LLC).

### Statistical analysis

Descriptive statistics, including means and standard deviations, were calculated. Standard deviation scores (z-scores) for height were calculated using standard growth measures from the World Health Organization (WHO) as comparators [35, 36]. All statistical analyses were performed using Stata MP v15.1 (College Station, TX, USA).

## Results

### Medical history and demographics

The clinical manifestations and *GALNS* mutations identified in the 21 MPS IVA patients (15 females, 6 males; mean age 15.5 ± 8.1 years, range 3.4–30.9 years) in this study are shown in Tables 1 and 2. Based on an estimated 14.7 million live births from 1985 to 2013, we estimate the national birth prevalence of MPS IVA in Malaysia to be 1 per 701,000 live births. Patients were mostly aged younger than 20 years (71%) and 57% were Malay, 29% were Chinese and 14% were Indian. This distribution of ethnic backgrounds is broadly in line with that of the Malaysian general population, which comprises 68.6% local ethnic groups of mostly Malays, 23.4% Chinese and 7% Indian [37]. All patients were still being followed up at time of writing except for patient 15, who passed away at the age of 18.5 years due to respiratory failure associated with a lung infection.

**Table 1:** Clinical and molecular characteristics of Malaysian MPS IVA patients

Pt	Sex	Ethnicity	Age at first symptoms, years	Age at diagnosis, years	Current age, years	Height, cm	Height z-score	6-MWT, metres	Assistive device	*GALNS* gene mutation	
										1st Allele	2nd Allele
1	F	Malay	0.2	2	3.4	91	−1.81	NP	None	c.502G>T	c.503G>T
2	F	Malay	1	1.7	3.4	78.8	−4.85	NP	None	c.473_477delAGTGG	c.1364+1G>A
3	F	Malay	2	4	4	85.8	−3.93	246.95	None	c.647T>C	c.958G>A
4	F	Malay	4	6	6	97	−3.54	300	None	c.812T>C	c.950G>A
5	M	Indian	2	3.9	8.8	90	−6.99	183	None	c.235T>C	c.235T>C
6	M	Malay	2	4	9.3	85.3	−7.98	296	None	c.1523T>C	c.812T>C
7^a^	F	Chinese	5	6.2	10.1	97	−6.58	96	Wheelchair	c.953T>G	c.106_111delCTGCTC
8	F	Malay	2	5	12.3	88	−9.44	278	Wheelchair	c.463G>A	mutation not found
9^b^	F	Malay	0.5	5.7	14	95.5	− 9.26	NP	None	NP	NP
10	F	Chinese	1	2	15.5	92.5	−10.20	219	Wheelchair	c.398A>C	c.106_111delCTGCTC
11^b^	F	Malay	1	8	16	97	−9.65	NP	Wheelchair	NP	NP
12^a^	F	Chinese	3	6	16.7	89	−10.98	50	Wheelchair	NP	NP
13	M	Indian	3	7.8	16.8	124	−6.62	50	Walker	c.218A>G	c.551G>A
14	F	Chinese	2	11	17.4	93	−10.51	21.5	Walker, Wheelchair	c.346G>A	mutation not found
15^c^	F	Malay	1	10	18.3(died)	85	−11.86	Cannot walk	Wheelchair	NP	NP
16^a^	F	Chinese	6	7.7	21.6	99.5	−9.73	48	Wheelchair	c.953T>G	c.106_111delCTGCTC
17^d^	M	Malay	6	12	22.5	116	−8.29	Cannot walk	Wheelchair	c.953T>G	mutation not found
18	M	Indian	2	18	25	97	−10.9	Cannot walk	Wheelchair, BIPAP	c.647T>C	c.647T>C
19^c^	M	Malay	1	3	26.4	87	−12.3	Cannot walk	Wheelchair	c.473_477delAGTGG	c.1364+1G>A
20	F	Chinese	2	4	26.9	92.8	−10.76	259.2	Walker, Wheelchair	c.512A>C	c.106_111delCTGCTC
21^d^	F	Malay	8	17	30.9	83	−12.25	50.7	Wheelchair	c.953T>G	mutation not found

Current age, denotes age at recruitment

^a, b, c, d^, denotes respective siblings

*Abbreviations*: *6-MWT* 6-min walk test; *BIPAP* bi-level positive pressure breathing assistance; *del* Deletion, *F* Female, *M* Male, *NP* Not performed, *Pt* Patients

**Table 2:** Patient demographic, first symptoms and anthropometric data

Demographic	Number of patients	
Total patients	21 (16 families)	
Female	15 (71%)	
Male	6 (29%)	
Ethnicity		
Malay	12 (57%)	
Chinese	6 (29%)	
Indian	3 (14%)	
Age at first symptoms	2.6 ± 2.1 (years, mean ± SD)	
Age at diagnosis	6.9 ± 4.5 (years, mean ± SD)	
First symptoms as reported by carers		
Pectus carinatum	12 (57%)	
Knock knees	9 (43%)	
Kyphosis	6 (29%)	
Short stature	6 (29%)	
Lax wrists	2 (10%)	
Abnormal gait	1 (5%)	
Falls	1 (5%)	
Current age, at recruitment	15.5 ± 8.1 (years, mean ± SD)	
Current height by age category, years	Mean, cm (min, max)	Z-score mean (min, max)^a^
0–4 (*n* = 3)	85.2 (78.8, 91)	−3.5 (−4.8, −1.8)
5–9 (*n* = 3)	90.8 (85.3, 97)	−6.2 (−8, −3.5)
10–14 (*n* = 3)	93.5 (88, 97)	−8.4 (−9.4, −6.6)
15–19 (*n* = 6)	96.8 (85, 124)	−10 (− 11.9, − 6.6)
≥ 20 (*n* = 6)	95.8 (83, 116)	− 10.7 (− 8.3, − 12.3)

*Abbreviations*: *max* Maximum, *min* Minimum, *SD* Standard deviation, *WHO* World Health Organization

^a^WHO growth charts used as reference population

The mean ages at first reported symptoms and confirmed diagnosis were 2.6 ± 2.1 and 6.9 ± 4.5 years, respectively. The most common first symptoms reported by carers were pectus carinatum (57%), followed by knock knees (43%; Table 2). Seven patients (33%), all aged less than 15 years, used no assistive device for walking, while the remainder used a wheelchair and/or a walker (67%). One patient (5%) used a bi-level positive pressure (BIPAP) breathing device.

**Fig. 1:**
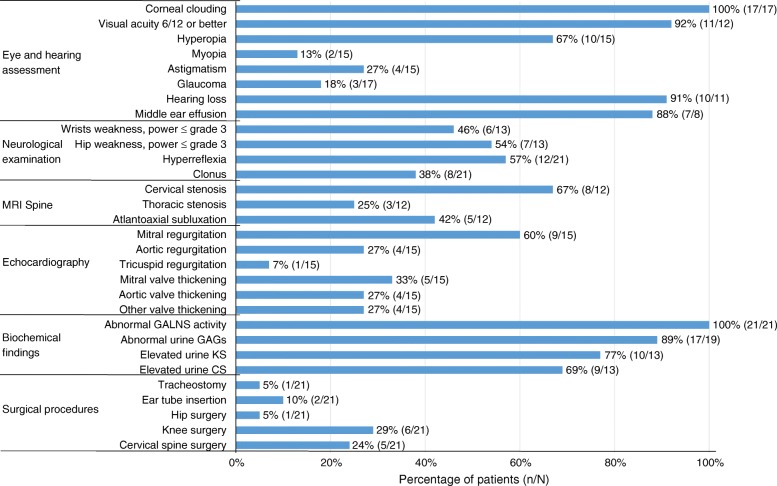
Frequency of clinical impairments, biochemical abnormalities and surgical procedures in Malaysian MPS IVA patients. Abbreviations: CS, chondroitin-6-sulfate; GAG, glycosaminoglycans; GALNS, galactose-6-sulfatase; KS, keratan sulfate; MRI, magnetic resonance imaging

Eight patients (38%) had undergone at least one surgical intervention, occurring before the age of 14 years. Knee surgery, performed in six patients (29%), was the most common surgical procedure, followed by cervical spine surgery (24%) (Fig. 1).

### Results of physical examinations and MRI spine

All our patients had the classical physical features described for MPS IVA – pectus carinatum, genu valgum and hypermobile wrist joints – although no formal assessments of joint range of motion were performed. All patients had significant short stature with mean z-scores ranging from −3.5 for those aged 4 years and younger, to −10.7 for those older than 20 years of age (Table 2). All the patients in our study (100%) had the severe phenotype. Approximately half the patients (57%) had documented hyperreflexia and 38% had clonus, indicating upper motor neuron lesion (Fig. 1). Thirteen patients had muscle power documented. Muscle weakness of MRC grade 3 and below was noted in wrist extension and flexion for six patients (46%) and in hip flexion for seven patients (54%). Handgrip weakness was noted in most patients although no formal assessment was performed. Spinal magnetic resonance imaging (MRI) data for 12 patients showed that eight patients (67%) had cervical stenosis, three patients (25%) had thoracic stenosis and five patients (42%) had atlantoaxial subluxation.

### Endurance and respiratory function

The patients displayed limited endurance and respiratory function. Thirteen patients completed a 6-MWT (Table 1). Of the eight patients who did not perform the 6-MWT, two were less than 4 years old and did not cooperate, two were post-operative and four – all older than 18 years – could no longer walk. Shorter mean walking distances were noted with increasing age (Table 3). Spirometry data were available for 8 patients; mean FVC was 0.6 L (SD 0.1) in patients 18 years and younger, and 0.9 L (SD 0.2) in patients older than 18 years. Four patients underwent overnight pulse oximetry monitoring and all showed significant desaturations during sleep (results not shown) but none underwent a formal sleep study to confirm possible obstructive sleep apnoea.

**Table 3:** 6-MWT and FVC in Malaysian MPS IVA patients by age group compared with other studies

	Mean ± SD		
	This study	Harmatz et al. [15]	Lin et al. [16]
6-MWT			
0–4 years old	246.9 m (*n* = 1)	251.6 ± 121.5 m (*n* = 37)	–
5–11 years old	218.8 ± 98.2 m (*n* = 4)	232.5 ± 140.1 m (*n* = 127)	–
12–18 years old	123.7 ± 116.4 m (*n* = 5)	181.2 ± 177.3 m (*n* = 84)	–
> 18 years old	119.3 ± 121.2 m (*n* = 3)	193.1 ± 148.5 m (*n* = 68)	–
Overall 6-MWT^a^	161.4 ± 110.1 m (*n* = 13)	212.6 ± 152.2 m (*n* = 316)	235.3 ± 125.5 m (*n* = 11)
Overall age	15.5 ± 8.1 years	14.5 years	12.6 ± 6.6 years
Spirometry, FVC			
≤ 18 years old	0.6 ± 0.1 L (*n* = 5)	1.1 ± 0.7 L (*n* = 256)	–
> 18 years old	0.9 ± 0.2 L (*n* = 3)	1.5 ± 1.1 L (*n* = 69)	–

*Abbreviations*: *6-MWT* 6-min walk test, *FVC* Forced vital capacity, *SD* Standard deviation

^a^ Only patients who completed the test were included

### Cardiology

Fifteen patients underwent echocardiography. The most frequently observed cardiac abnormality was mitral regurgitation (60%; Fig. 1), followed by thickened mitral valve (33%), thickened aortic valve (27%) and aortic regurgitation (27%). Mean ejection fraction was 58.5% (SD 24.9).

### Ear and eye examinations

Of the patients who underwent audiometry assessment, almost all (10/11, 91%) had some form of hearing loss. Middle ear effusion was present in seven out of eight patients assessed (88%; Fig. 1). Seventeen patients underwent complete ocular assessment by ophthalmologist. Of these, all (100%) had corneal clouding, but none needed corneal transplantation, three (18%) had glaucoma and none had pigmentary retinopathy or optic disc changes. Hyperopia was the commonest type of refractive error (67%) among the 15 patients who underwent cycloplegic refraction. Eleven of the 12 patients (92%) who underwent visual acuity testing had documented vision of 6/12 or better (Fig. 1).

### Urine GAGs and GALNS enzyme analysis

All our patients had reduced GALNS activity (Fig. 1 and Additional file 1: Table S1). Elevated urine GAGs were detected in 89% of patients, but only 77% and 69% were found to have elevated urine KS and CS, respectively.

### *GALNS* gene mutations

A total of 30 mutant alleles were identified in 17 patients (88.2%) from 15 different families and the remaining four alleles were unknown (Table 1). The mutant alleles belonged to 18 distinct genotypes: 13 missense, three nonsense, one small deletion and one splice site mutation (Table 4). Of these, eight were novel mutations (Tyr133Ser, Glu158Valfs*12, Gly168*, Gly168Val, Trp184*, Leu271Pro, Glu320Lys, Leu508Pro). Six recurrent mutations (Leu36_Leu37del, Glu158Valfs*12, Phe216Ser, Leu271Pro, Met318Arg, c.1364+1G>A) were identified in unrelated patients. Two mutations, Cys79Arg and Phe216Ser, were found homozygous in patients 5 and 18, respectively (Table 1). Patients 5, 15, 18 and 19 had parental consanguinity.

**Table 4:** Mutations identified in the *GALNS* gene of 17 Malaysian patients

Nucleotide change	Amino acid change	Exons/ IVS	Allele frequency, *N* = 34 (%)	Reference
c.106_111delCTGCTC	p.(Leu36_Leu37del)	1	4 (12%)	Yang, 2001 [38]; Wang 2010 [39]
c.218A>G	p.(Tyr73Cys)	2	1 (3%)	Lee, 2012 [17]
c.235T>C	p.(Cys79Arg)	2	2 (6%)	Bidchol, 2014 [40]
c.346G>A	p.(Gly116Ser)	4	1 (3%)	Tomatsu, 2004 [41]
c.398A>C	p.(Tyr133Ser)	4	1 (3%)	novel
c.463G>A	p.(Gly155Arg)	5	1 (3%)	Bunge, 1997 [42]
c.473_477delAGTGG	p.(Glu158Valfs*12)	5	2 (6%)	novel
c.502G>T	p.(Gly168*)	5	1 (3%)	novel
c.503G>T	p.(Gly168Val)	5	1 (3%)	novel
c.512A>C	p.(Asp171Ala)	5	1 (3%)	Sukegawa, 2000 [43]
c.551G>A	p.(Trp184*)	5	1 (3%)	novel
c.647T>C	p.(Phe216Ser)	7	3 (9%)	Morrone, 2014 [44]
c.812T>C	p.(Leu271Pro)	8	2 (6%)	novel
c.950G>A	p.(Gly317Glu)	9	1 (3%)	Caciotti, 2015 [45]
c.953T>G	p.(Met318Arg)	9	4 (12%)	Ogawa, 1995 [46]
c.958G>A	p.(Glu320Lys)	9	1 (3%)	novel
c.1364+1G>A	Skipping of exon 12	IVS 12	2 (6%)	Bunge, 1997 [42]
c.1523T>C	p.(Leu508Pro)	14	1 (3%)	novel

*Abbreviation*: *IVS* Intervening sequence

**Fig. 2:**
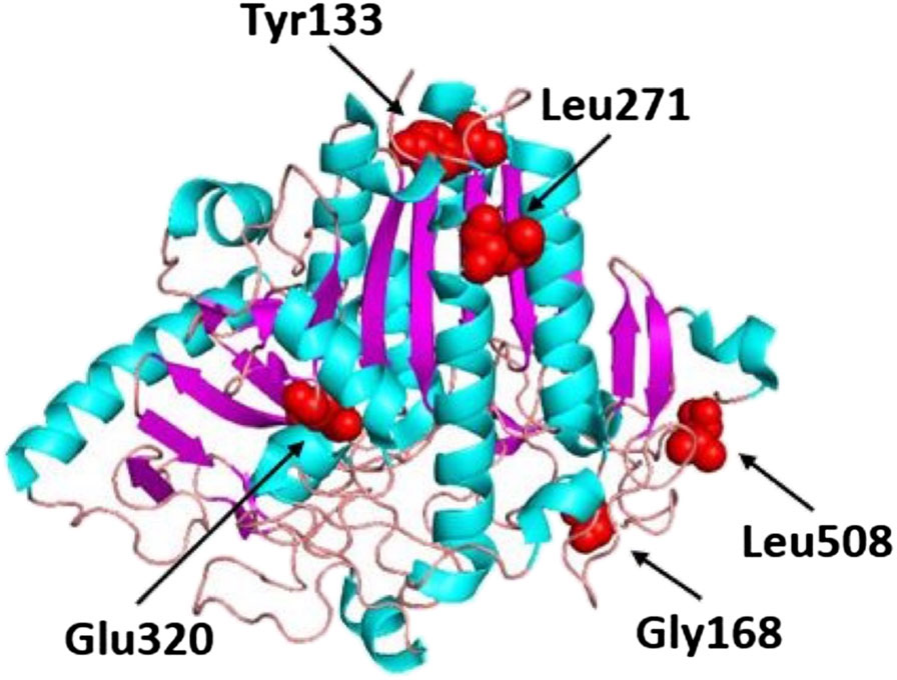
Location of five novel missense mutations mapped onto the protein structure of the human GALNS protein. Red indicates location of labelled mutations; alpha helices are indicated in cyan, beta sheets in magenta

Novel mutations were predicted to be pathogenic by all four in silico programs except for Leu271Pro, which was predicted to be pathogenic by only M-CAP and Condel (Additional file 1: Table S2). Four of the five novel missense mutations (Tyr133Ser, Gly168Val, Leu271Pro, Glu320Lys) affected domain 1 of the human GALNS glycoprotein and the remaining Leu508Pro mutation affected a C-terminal meander region (Fig. 2) [47]. Leu271 is located in an α-helix, so substitution to a proline residue would introduce a helix kink that perturbs the overall folding of the protein. Leu508 is located next to Cys507, a residue involved in forming a disulfide bridge with Cys501. Substitution of the small leucine residue to a bulkier proline residue would likely disturb the formation of this important disulfide bridge leading to destabilization of the protein. Since the remaining three mutations are located on the linker/loop region, comparisons of the wild type and mutant protein structures did not reveal any perceivable impact. However, the substitutions could affect the flexibility and local conformation of the protein.

## Discussion

This study is the first report to describe the clinical, biochemical and genetic profiles of all patients with confirmed MPS IVA in Malaysia; it showed that MPS IVA affects all major ethnicities in Malaysia.

The study of MPS IVA patients in Taiwan by Lin and colleagues noted a diverse phenotypic severity, as did the international study by Montaño and colleagues in which 25% of patients had a mild or intermediate phenotype [14, 16]. All the MPS IVA patients in our study had the severe phenotype. Similarly, Lee and colleagues in Korea reported nine of 10 subjects had the severe phenotype [17]. Our estimated birth prevalence for MPS IVA of 1 per 701,000 live births is lower than those previously reported [8–11]; under-diagnosis of attenuated phenotypes may have contributed to this discrepancy. This highlights the need for increased awareness of MPS IVA, especially of non-classical symptoms in those with attenuated disease, such as hip stiffness and pain without severe short stature [12]. The mean ages at initial symptoms and confirmed diagnosis reported in our study (2.6 and 6.9 years, respectively) are similar to those reported by Montaño et al. (2.1 and 4.7 years), and Lin et al. (2.0 and 5.7 years) [14, 16]. Common initial presenting symptoms seen in Malaysian patients – pectus carinatum, knock knees and kyphosis – are also similar to those observed in Taiwanese MPS IVA patients [16].

The prevalence of surgical interventions in the Malaysian MPS IVA patients was higher than that observed by Lin et al. (33% any surgical intervention, 13% undergoing spinal decompression) but similar to that observed in the study by Montaño et al. (51% of patients underwent spinal decompression) [14, 16]. Harmatz and colleagues reported a surgery prevalence of 71% in an MPS IVA population with an average age of 14.5 years [15]. The variable prevalence of surgical interventions among MPS IVA patients in these studies may be due to the diverse phenotypic severity but it may also reflect differing surgical practices in each country or region. For example, indication for surgery is less straightforward for prophylactic cervical fusion and/or decompression at an early age highlighting the need for more studies on timing of surgery and long term outcomes [3].

The limitations in endurance seen in our study appear to be more severe than those previously reported by Harmatz et al. [15] and Lin et al. [16] (Table 3). This may be explained by the older mean age and the severe phenotype of our study cohort. Nineteen percent of our patients can no longer walk. Mobility limitations in patients with MPS IVA may be due to atlantoaxial subluxation, progressive spinal compression, reduced respiratory function [14, 15] and progressive debilitating genu valgum [48]. This highlights the importance of monitoring and surgical interventions for the spine and lower limbs.

Our patients also had lower FVC values than those reported by Harmatz et al. [15]. This may be because of the severe phenotype as FVC volumes are affected by reduced height, malformed thorax and reduced upper airway patency [15]. Cardiac abnormalities, in particular mitral regurgitation and thickened mitral valve, were common in our MPS IVA population. Similar findings were reported in Taiwanese MPS IVA patients, where 45% of patients had mitral regurgitation and 32% had mitral stenosis [16], and in a study of German MPS IVA patients, where 28/54 (52%) of patients had valve thickening [49].

Many patients in our study had wrist joint weakness and laxity, a defect that is highly prevalent in MPS IVA patients [50]. The combination of reduced strength and wrist hypermobility greatly limits hand function and curtails many activities of daily living that involve gripping objects. Interventions aimed at maintaining wrist function may be valuable for improving patients’ abilities to perform daily activities independently.

All our patients had corneal clouding but none needed corneal transplantation, and most of the patients had visual acuity of 6/12 or better. Mild corneal clouding is typically reported in MPS IVA [51], although severe clouding has also been reported [52].

The mutational spectrum in the *GALNS* gene is highly heterogeneous among patients with MPS IVA in Malaysia. Nine of 14 coding exons were found to harbour disease-causing mutations. Despite this heterogeneity, we found that exon 5 was the most commonly mutated region, followed by exons 9 and 1. Mutations in these three exons accounted for more than half (51%) of the mutant alleles identified. These potential hotspot regions could facilitate the design of targeted molecular assays for rapid screening of mutations in the *GALNS* gene in Malaysian patients with MPS IVA. The second disease-causing mutation could not be identified in 23.5% of our patients, and this proportion is comparable to previous reports [44, 53]. This may be due to the presence of mutations, such as large deletions [45] or mutations within deep intronic regions [54], that are not detectable by sequence analysis. Therefore, incorporating other techniques, such as mRNA analysis and copy-number variation (CNV) assays, into the testing workflow for *GALNS* gene analysis could improve the diagnostic yield.

We found that missense mutation is the most common mutation type in the *GALNS* gene, and this matches the trend observed in HGMD [23]. One of the 10 most frequently reported mutations in the *GALNS* gene (Met318Arg) was found to be recurrent in our patients, and this is consistent with the high prevalence of this mutation among patients from the East and Southeast Asian region [55]. The use of variant data from 100 Singaporean Malays and 38 Singaporean Indians will enable identification of population-specific rare variants, and the availability of genetic data from an even larger cohort of this underrepresented population will further enhance our understanding of the genetic basis of this disease [56]. Based on the *GALNS* mutation reporting guidelines proposed by Morrone and colleagues [44], 14 of 18 (77.7%) distinct mutations in our patient cohort could be considered disease-associated, either because the mutation was identified in multiple unrelated patients or the predicted impact of the mutation on the protein was clear-cut. The remaining four missense mutations (Tyr133Ser, Gly168Val, Glu320Lys, Leu508Pro) were predicted to be pathogenic by all four in silico programs used and were considered likely to be disease-associated due to the presence of disease-associated mutations in trans [44]. The novel mutations identified in this study will be useful for assessing the diagnostic status of individuals related to MPS IVA patients and for genetic counselling purposes.

Our study is limited by incomplete data for many subjects, variable assessment of clinical and biochemical parameters, and the retrospective nature of the analysis. The lack of data on quality of life and activities of daily living also limits assessment of phenotypic severity. The number of patients is small, as is typical for a rare disease, and it is possible that attenuated phenotypes have not been diagnosed.

## Conclusion

Our study has added to the understanding of the natural history, phenotype and genotype of MPS IVA by evaluating all confirmed patients in Malaysia. All subjects in the study had the severe phenotype with significant clinical impairments. Regular evaluations and management according to the latest guidelines may improve their quality of life. Establishment of a national patient registry could capture data that can further optimize care and improve our understanding of this disease. In the longer term, the creation of a national centre of expertise for the diagnosis and management of MPS IVA patients may help consolidate and concentrate the diverse fields of medical expertise required to treat these patients. Clinical findings, GALNS enzyme activity testing and molecular analysis are essential for precise diagnosis and prognosis of MPS IVA.

## Additional file

Additional file 1: **Table S1.** Biochemical characteristics of Malaysian MPS IVA patients. **Table S2.** In silico prediction of novel missense mutations in the *GALNS* gene. (DOCX 17 kb)

## Data Availability

All data generated or analysed during this study are included in this published article (and its supplementary information files).
