# Supplementary material for: Clinical, biochemical and genetic profiles of patients with mucopolysaccharidosis type IVA (Morquio A syndrome) in Malaysia: the first national natural history cohort study
Source: Orphanet J Rare Dis. 2019 Jun 14;14:143. doi: 10.1186/s13023-019-1105-6 (PMC6570902; doi:10.1186/s13023-019-1105-6)
Supplement: Supplementary file 1 — Table S1. Biochemical characteristics of Malaysian MPS IVA patients. Table S2. In silico prediction of novel missense mutations in the GALNS gene. (DOCX 17 kb) [file 13023_2019_1105_MOESM1_ESM.docx]

Additional file 1

Table S1 Biochemical characteristics of Malaysian MPS IVA patients

| **Pt** | **Urine GAGs*, g/mol creatinine** | **Urine KS/CS*** | **Urine CS*** | **GALNS activity,**  **nmol/mg protein/17 hours,**  **(Reference 76–360)** |
| --- | --- | --- | --- | --- |
| 1 | 56.13 ­↑ | ↑­ | ↑ | 10 |
| 2 | 42.8 ­↑ | ­↑ | ↑ | <0.1 |
| 3 | 17.53 <> | <> | <> | 0.59 |
| 4 | 19.71 ­↑ | <> | <> | <0.1 |
| 5 | 30.62 ­↑ | ↑ | ↑ | 51 |
| 6 | 26.64 ­↑ | ­↑ | <> | 3.1 |
| 7^a^ | NP | NP | NP | <0.1 |
| 8 | 14.55 ­↑ | ↑ | ↑ | <0.1 |
| 9^b^ | 22 ­↑ | ↑ | ↑ | <0.1 |
| 10 | 31.33 ­↑ | ↑ | ↑ | <0.1 |
| 11^b^ | 19.06 ­↑ | ↑ | ↑ | 0.51 |
| 12^a^ | NP | NP | NP | 1 |
| 13 | 9.38 <> | NP | NP | 2.01 |
| 14 | 39.87 ­↑ | ↑ | <> | 1 |
| 15^c^ | 36.06 ­↑ | NP | NP | <0.1 |
| 16^a^ | ­↑* | NP | NP | 1 |
| 17^d^ | 9.75 ­↑ | NP | NP | 0.3 |
| 18 | 23 ­↑ | NP | NP | 1 |
| 19^c^ | 17.08 ­↑ | <> | ↑ | <0.1 |
| 20 | ­↑* | ↑­ | ↑ | 0.68 |
| 21^d^ | 9.32 ­↑ | NP | NP | 0.32 |

*Biochemical measurements were made in different laboratories with different assays and may not be comparable between patients. **↑** denotes elevated relative to reference, <> denotes not elevated relative to reference.

Abbreviations: CS, chondroitin-6-sulfate; GAGs, glycosaminoglycans; KS, keratan sulfate; NP, not performed; Pt, patients.

Table S2 *In silico* prediction of novel missense mutations in the *GALNS* gene

| Missense mutation | MutationTaster2 | | FATHMM-XF | | M-CAP | | Condel | |
| --- | --- | --- | --- | --- | --- | --- | --- | --- |
|  | P | Prediction | Score | Prediction | Score | Prediction | Score | Prediction |
| Tyr133Ser | 0.999999989541056 | Disease causing | 0.913156 | Pathogenic | 0.827 | Possibly pathogenic | 0.775700009753 | Deleterious |
| Gly168Val | 0.999999999999932 | Disease causing | 0.924891 | Pathogenic | 0.762 | Possibly pathogenic | 0.550217930102 | Deleterious |
| Leu271Pro | 0.99630857781939 | Polymorphism | 0.226008 | Benign | 0.588 | Possibly pathogenic | 0.642627807802 | Deleterious |
| Glu320Lys | 0.999999963876575 | Disease causing | 0.909103 | Pathogenic | 0.595 | Possibly pathogenic | 0.652614612683 | Deleterious |
| Leu508Pro | 0.999999999975515 | Disease causing | 0.934533 | Pathogenic | 0.706 | Possibly pathogenic | 0.565879658465 | Deleterious |
